# Core-Shell Magnetic Nanoparticles for Highly Sensitive Magnetoelastic Immunosensor

**DOI:** 10.3390/nano10081526

**Published:** 2020-08-04

**Authors:** Raffaele Campanile, Emanuela Scardapane, Antonio Forente, Carmine Granata, Roberto Germano, Rocco Di Girolamo, Antonio Minopoli, Raffaele Velotta, Bartolomeo Della Ventura, Vincenzo Iannotti

**Affiliations:** 1 Department of Physics “E. Pancini”, University of Naples Federico II, Via Cintia 26, I-80126 Napoli, Italy; raffaele.campanile@unina.it (R.C.); emanuela.scardapane@unina.it (E.S.); a.forente@studenti.unina.it (A.F.); antonio.minopoli2@unina.it (A.M.); rvelotta@unina.it (R.V.); bartolomeo.dellaventura@unina.it (B.D.V.); 2 PROMETE Srl, CNR Spin off, Piazzale Tecchio, 45 80125 Napoli, Italy; germano@promete.it; 3 Institute of Applied Sciences and Intelligent Systems of the National Research Council (CNR-ISASI), Via Campi Flegrei 34, I-80078 Pozzuoli, Italy; c.granata@isasi.cnr.it; 4 Department of Mathematics and Physics-University of Campania “L. Vanvitelli”, Viale Abramo Lincoln 5, 81100 Caserta, Italy; 5 Department of Chemistry, University of Naples “Federico II”, Via Cintia 26, I-80126 Napoli, Italy; rocco.digirolamo@unina.it; 6 Institute for Superconducting, Oxides and other Innovative Materials and Devices of the National Research Council (CNR-SPIN), Piazzale V. Tecchio 80, I-80125 Napoli, Italy

**Keywords:** core-shell magnetic nanoparticles, magnetoelastic biosensor, human IgG, photochemical immobilization technique

## Abstract

A magnetoelastic (ME) biosensor for wireless detection of analytes in liquid is described. The ME biosensor was tested against human IgG in the range 0–20 μg∙mL^−1^. The sensing elements, anti-human IgG produced in goat, were immobilized on the surface of the sensor by using a recently introduced photochemical immobilization technique (PIT), whereas a new amplification protocol exploiting gold coated magnetic nanoparticles (core-shell nanoparticles) is demonstrated to significantly enhance the sensitivity. The gold nanoflowers grown on the magnetic core allowed us to tether anti-human IgG to the nanoparticles to exploit the sandwich detection scheme. The experimental results show that the 6 mm × 1 mm × 30 μm ME biosensor with an amplification protocol that uses magnetic nanoparticles has a limit of detection (LOD) lower than 1 nM, works well in water, and has a rapid response time of few minutes. Therefore, the ME biosensor is very promising for real-time wireless detection of pathogens in liquids and for real life diagnostic purpose.

## 1. Introduction

In recent years biosensors have proven to be an interesting platform for developing sensitive and portable devices devoted to detecting biological or chemical entities for a variety of applications, such as monitoring of environmental pollutants, food and water safety, and biomedicine [1].

Biosensors are sensing devices that detect organic or inorganic compounds (target) with the help of specific biological recognition elements (bioreceptors) and produce a measurable signal due to an appropriate transducer system. The major advantages of biosensors are portability, ease of use, rapidity of response and low-cost. Compared to other robust and accurate laboratory techniques (e.g., enzyme-linked immunosorbent assay, ELISA [2] and polymerase chain reaction, PCR [3]), biosensors do not need expert personnel, well-equipped laboratories or complex sample pretreatments, which make them suitable for in-situ and real-time monitoring of contaminants.

Characteristics and applications of biosensors strongly depend on the choice of both biosensing elements and transducers. Examples of bioreceptors are enzymes, antibodies, nucleic acids, and aptamers. Regardless of recognition elements chosen, the functionalization step, i.e., the immobilization of bioreceptors on the surfaces of the transducer, has proven to be crucial for biosensors effectiveness [4]. Antibody-based biosensors, i.e., immunosensors, benefit from the natural high specificity of antigen-antibody interactions. The easiest way to immobilize antibodies (Abs) relies on non-covalent interplay (e.g., van der Waals or electrostatic forces) and gives rise to a resulting binding to the sensor surfaces which is weak (non-covalent) and randomly oriented. The procedures to overcome these limits are usually time-consuming, quite complex or can affect the antigen-specificity of Abs [5]. Complexity and delicacy of functionalization procedures usually compromise the detection speed of biosensors. 

Depending on the type of transducers, biosensors are classified in electrochemical [6,7], optical [8,9] and acoustic-wave biosensors [10,11]. Recently, among the magnetic biosensors [12,13,14], magnetoelastic materials (ME) have emerged as interesting acoustic-wave transducers for development of high-sensitive biosensors [15]. ME sensors can be placed in a vibration condition due to magnetostriction effect, at the characteristic resonance frequency $f_{0}$, employing time-varying magnetic fields. The attaching of a small mass to the surface of the material involves a shift of the resonance frequency, which can therefore be used as sensing parameter. In fact, the mass addition dampens the resonance behavior of the resonant sensor. The principal competitive advantage of ME biosensors is that they are wireless, namely there is no physical connection between the detection electronics and the sensor. Wireless sensing turns out to be a very interesting feature for applications as point of care testing, especially by considering that most of the other devices require complex wiring for power and measurement. Furthermore, ME transducers are composed by a low-cost raw material, and their compact size make them suitable for multiplexing schemes. However, sensitivity remains the main drawback of these transducers. Recent attempts to enhance sensitivity rely on several strategies such as the reduction of the size of ME platforms [16], and the amplification of the signal response by using gold nanoparticles [17]. It should be noted that the attempt of reducing the dimension of sensor platforms is limited by manufacturing difficulties and loss of the intensity of the signal that occur when the microscale is reached [18,19]. 

Gold nanoparticles (AuNPs) are widely used in biosensing due to their high chemical stability, biocompatibility and large specific surface area on which bioreceptors such as Abs can be properly immobilized. In the case of a ME sensor, the amplification of the response signal via AuNPs exploits the typical sandwich-assay scheme, in which AuNPs, functionalized with bioreceptors (e.g., Abs), bind to the target-bioreceptor pair (e.g., antigen-Ab) on the sensor surface. Thus, the mass loading on the ME sensor surface increases, enhancing its sensitivity of detection. 

Besides AuNPs, magnetic nanoparticles have also found application as signal labels in biosensing systems, such as molecular detection and related strategies that rely on ligand-receptor binding. In particular, iron oxide magnetic nanoparticles (NPs), such as magnetite (Fe_3_O_4_) and maghemite (γ-Fe_2_O_3_), are particularly appealing due to their magnetic properties, tunable size, biocompatibility, and greater ease of synthesis than other magnetic materials [20,21,22]. Indeed, recent studies have shown how to use magnetic nanoparticles to improve the efficiency of the functionalization process [23], to realize multiplexing immunoassays [24] and for magnetic detection [14,25,26]. 

In this paper, we describe how core-shell magnetic nanoparticles (Fe_3_O_4_@Au, i.e., gold nanoflower grown on a magnetic core) can be used to amplify the signal from a ME biosensor for wireless detection of contaminants in liquid. The use of the magnetic properties of nanoparticles is a current and relevant topic for scientific community, although the tendency to aggregation is an obstacle to their applications. In our case, the Fe_3_O_4_ NPs gold coating and functionalization protocols employed for signal amplification have also the effect to prevent nanoparticles aggregation, so that other complex and time-consuming techniques can be avoided [27,28]. The sensor platform is a low-cost and commercially available ME material (Metglas 2826), shaped in a ribbon of small size (6 mm × 1 mm × 30 μm). We propose the synergy of two strategies to increase the performance of this sensor: (i) a new amplification procedure that exploits gold coated nanoparticles with magnetic core (ii) the use of reliable, quick and easy-to-use antibody functionalization procedure. We observed that the signal amplification obtained with gold coated nanoparticles with magnetic core (Fe_3_O_4_@Au) was significantly higher than that obtained using AuNPs. The fact that in our case both the magnetic sensitive platform and magnetic nanoparticles contribute to the measurements process is an element of novelty in comparison with standard configurations widely discussed in literature [29,30]. It should be noted that the amplification of the shift of the resonance frequency of the ME sensor is due to the mass of magnetic nanoparticles and does not depend directly on their magnetic properties. Indeed, the advantage of the magnetic core of the nanoparticles relies in its coupling with the local magnetic field, which in turn leads to an increase of the local density at proximity of the ribbon surface. Such an effect can be well understood by working out the magnetic field produced by the magnetized ribbon and comparing its action on a magnetic nanoparticle with the thermal energy (Brownian motion).

Regarding the functionalization procedure, the photochemical immobilization technique (PIT) recently introduced by Della Ventura et al. [31] not only was used for the first time to functionalize a ME material (coated with gold), but also the gold nanoflowers grown on the magnetic nanoparticles. This technique, based on a controlled UV-activation of Abs, has proven to be an effective [6,8,10,32,33,34] and competitive methodology since it is rapid and user-friendly and leads to strong (covalent) and conveniently oriented bonds of Abs on the sensor surfaces, without affecting the intrinsic selectivity of the antibodies.

The experimental results show that the proposed ME biosensor has a reliable stability in liquid, a quick response to antigen exposure and exhibits a limit of detection (LOD) lower than 1 nM.

## 2. Principles of ME Biosensor

ME biosensors operating principle is based on the Joule magnetostriction of magnetic materials, which can vibrate longitudinally at a characteristic frequency, depending on physical parameters of the materials, when subject to a time varying magnetic field [15]. For a ribbon shaped sensor, that under the above conditions undergoes a plane-stress or biaxial state, the fundamental resonance frequency in air, $f_{air}$, is given by equation:

(1) $$f_{air}=\frac{1}{2L}\sqrt{\frac{E}{\rho \left(1-\nu \right)}},$$

where *L* is the length of the ribbon, *E* its Young’s modulus, ρ its density and ν the Poisson’s ratio [35], while in low viscosity liquids the resonance frequency, $f_{0}$, is given by equation:

(2) $$f_{0}=f_{air}-\frac{\sqrt{\frac{\eta_{liq}\rho_{liq}}{\pi }}}{2\rho d}\;\;\cdot \sqrt{f_{air}},$$

where $\eta_{liq}$ and $\rho_{liq}$ are the dynamic viscosity and density of the liquid. The relationship (1) is obtained by approximating the ribbon to a thin beam, a valid approximation when the thickness is negligible with respect to the other two dimensions involved. The length to width ratio is needed to be greater than five for a good magnetoelastic response of the material, the latter being further improved for ratios greater than fourteen [36]. In our case we chose a length to width ratio of six because, as the length and mass of the sensor increases, the mass sensitivity $\left(S_{m}\right)$ decreases [37]. When the testing temperature, humidity and other environmental parameters are constant, the resonance frequency change of the magnetoelastic sensor depends only on the mass change on its surface. In the approximation of small mass loading (Δ*m* « *M_ME_*) uniformly distributed on the ribbon surface, the shift in resonance frequency in low viscosity liquids is given by equation:

(3) $$\Delta f_{0}=-\frac{5f_{0}-3f_{air}}{4M_{ME}}\Delta m,\;\;$$

where $f_{0}$ is the initial resonance frequency in liquid, $\Delta f_{0}$ its variation due to the detection of antigens of mass Δ*m* and $M_{ME}$ is the initial mass of the ribbon [37]; thus, the sensitivity of the sensor is:

(4) $$S_{m}=-\frac{∆f_{0}}{∆m}=\frac{5f_{0}-3f_{air}}{4M_{ME}}.\;$$

## 3. Materials and Methods

### 3.1. ME Sensors Fabrication

ME sensors platforms, composed of Metglas alloy 2826 (Fe_40_Ni_40_P_14_B_6_), were purchased from Honeywell Corporation (Morriston, NJ, USA) in the form of roll and cut in ribbon form with the dimensions 6 mm × 1 mm × 30 μm using a computer-controlled laser cutting machine. The ME ribbons were ultrasonically cleaned sequentially in ethanol and distilled water each for 20 min, then dried in an inert atmosphere.

The surfaces of the cleaned ME ribbons were covered with a layer of titanium (Ti) in thickness of 30 nm, followed by a layer of gold (Au) in thickness of 100 nm. The titanium inner layer was used to improve the adhesion of the gold film on the sensor surfaces, while the gold layer was exploited to enhance the immobilization process of sensing-elements (i.e., antibodies in this study) on the sensor surfaces and also to protect the ME ribbons from corrosion. 

Compared with other works found in literature [38,39], polishing and annealing of the ME ribbons were not needed in our procedure.

### 3.2. Antibodies Immobilization

The ME sensor surfaces coated with gold and washed sequentially in ethanol and ultrapure water were functionalized by means of antibodies (Abs) human IgG produced in Goat, purchased by ImmunoReagents Inc. (Raleigh, NC, USA). 

The adopted functionalization procedure was the photochemical immobilization technique (PIT) [31], a powerful and quick methodology based on an appropriate UV-activation of Abs, whose effectiveness was already confirmed in several application for biosensing [6,8,10,32,33,34]. It was demonstrated that this method leads at the same time to a strong (covalent) binding of Abs onto gold surfaces while orienting Abs with one fragment antigen-binding site (Fab) exposed to the solution. As an immediate consequence, the antigen detection efficiency of the immunosensor is enhanced. The functionalization procedure via PIT involved the following steps: the ME sensor was mounted into a fluidic circuit and immersed in MilliQ water; a quartz cuvette containing 1 mL of Abs dissolved in ultrapure water (25 µg∙mL^−1^) was irradiated by UV light (lamp Trylight^®^) for 30 s, which is the optimal irradiation time for PIT; since the Abs binding sites remain active for about five minutes, immediately after the irradiation, the activated Abs solution was placed in the fluidic circuit and conveyed onto the ME sensor surfaces. The solution flowed into the closed fluidic circuit for several minutes. 

In this study gold nanoparticles (AuNPs) and core-shell (Fe_3_O_4_@Au) NPs were used to amplify the biosensor response, in order to determine, for the same mass of the nanoparticles, whether the magnetic action produces an improvement in the sensitivity of the biosensor. The functionalization was again achieved by PIT for both types of NPs. A volume of 1 mL of suspended NPs in MilliQ water was prepared, whereas a volume of 100 µL of Abs solution (25 µg∙mL^−1^), irradiated by UV-light for 30 s, was added in twenty spikes (5 µL each) to the NPs solution and gently stirred in order to avoid aggregation. The absorbance spectra of the functionalized NPs, characterized by the UV/vis spectrophotometer (model 6715 Jenway, Cole-Parmer^®^ Company, Illinois USA), showed a red-shift of 3 nm of the LSPR wavelength, in accordance with the change of both types of NPs refractive index due to immobilization of antibody onto gold layer [33].

### 3.3. Synthesis of AuNPs

The AuNPs were synthesized by chemical reduction of tetrachloroauric (III) acid trihydrate (HAuCl_4_·3H_2_O) through sodium citrate (Na_3_C_6_H_5_O_7_) [40]. A solution of 50 mL of ultrapure water and 0.5 mL solvated HAuCl_4_·3H_2_O (24 mM) was heated up at 150 °C and stirred constantly. Afterwards, 6 mL of sodium citrate dihydrate (39 mM) was added into the boiling solution to achieve particle nucleation. 

To further increase particle growth, another 4.2 mL of HAuCl_4_·3H_2_O (24 mM) was added after 2 min. The color of the solution changed from transparent to black to finally move to bright red in few minutes. As final step, the solution was let cool down for 2 h keeping the same stirring. 

In order to employ AuNPs as signal amplification factor, it was necessary to remove the sodium citrate, in which they were suspended to avoid aggregation, during the functionalization of the surface with Abs. The centrifuge protocol working conditions for 1 mL of citrate AuNPs (the dilution: 200 μL of citrate AuNPs and 800 μL of ultrapure water) was achieved through two steps: (a) 15 min at 9000× g, and (b) 10 min at 5000× g. After each centrifugation, the pellet was re-suspended in 1 mL ultrapure water. 

The resulting optical density (OD) was ≃1.0 that corresponds to ≃10^11^ AuNPs·mL^−1^ with diameter of 40 nm [41].

### 3.4. Au Coating of Fe_3_O_4_ Nanoparticles

The magnetic Fe_3_O_4_ nanoparticles (average diameter 30 nm) were purchased by MERCK (catalog number 747327) and gold coated as follows. 100 μL of magnetic nanoparticles were added to a solution containing 50 mL of MilliQ water and sodium citrate (10 mg∙mL^−1^) and they were heated until 90 °C with vigorous stirring. Once the temperature was reached, 50 μL of HAuCl_4_·3H_2_O (10 mg∙mL^−1^) was added to the solution for four times every ten minutes. At this point the solution was let to cool down until it reached the room temperature keeping the same stirring. As a result of such a procedure a colloidal solution of 50 mL of Fe_3_O_4_@Au NPs was obtained.

### 3.5. Characterization of Nanoparticles

Transmission electron microscopy (TEM) micrographs were collected using a FEI Tecnai G2 S-twin apparatus (University of Naples Federico II, Italy) operating at 200 kV (LaB6 source). The particle powder samples were transferred on carbon-coated copper grids (200 mesh) by dispersing them in ethanol and then adding one drop on the copper grid and evaporating the solvent. 

**Figure 1:**
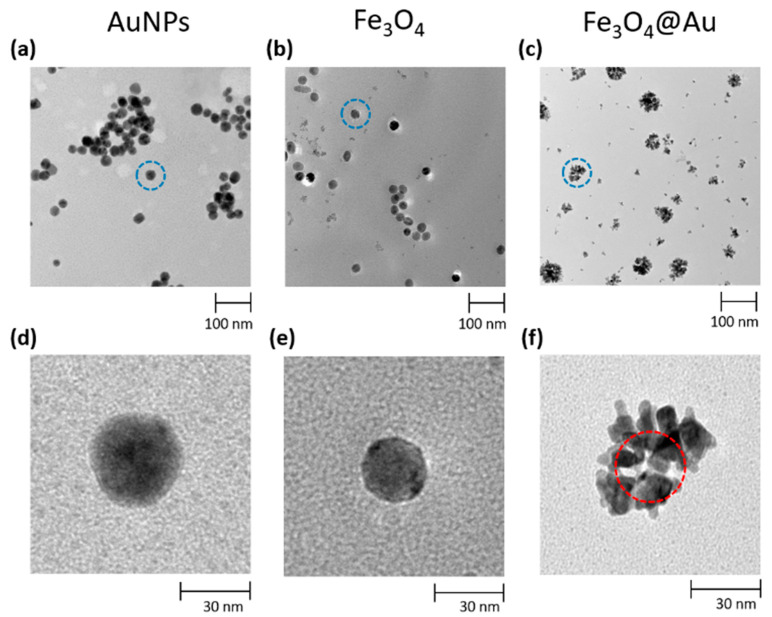
TEM micrographs of (**a**) AuNPs, (**b**) Fe_3_O_4_ NPs and (**c**) Fe_3_O_4_@Au core-shell NPs (gold nanoflower grown on a magnetic core). In the lower part of the figure the circled portions in panels (**a**,**b**,**c**) are respectively reported in detail in panels (**d**,**e**,**f**). In panel (**f**) the magnetic core is highlighted with a red circle.

Figure 1 shows the TEM micrograph of AuNPs synthetized according to the abovementioned protocol (a) as well as the Fe_3_O_4_ magnetic nanoparticles before (b) and after (c) the gold coating (Fe_3_O_4_@Au core-shell NPs).

**Figure 2:**
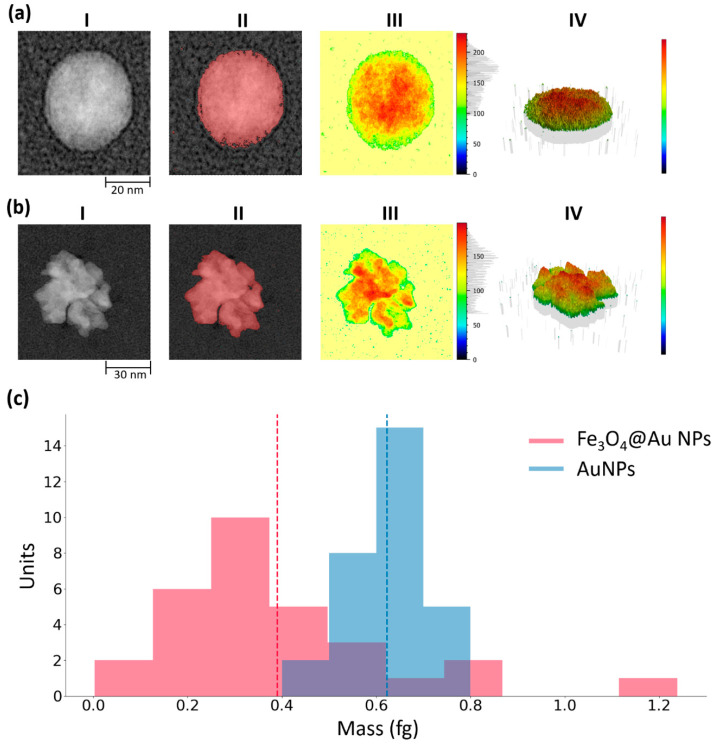
The steps of the protocol employed to estimate the mass of AuNPs (**a**) and Fe_3_O_4_@Au NPs (**b**) using the software SPIP Mountains 8. The nanoparticle of interest was considered and extracted from the collective TEM micrograph (**I**), the contour of the nanoparticles was detected and distinct by the background (**II**), then particle surface (**III**) and its 3D rendering (**IV**) were generated. The latter was employed to estimate the volume of the object. (**c**) Mass distribution of AuNPs and Fe_3_O_4_@Au NPs (core-shell NPs). The mean and the standard deviation for the mass distributions were 0.4 ± 0.2 fg for Fe_3_O_4_@Au NPs and 0.63 ± 0.09 fg for AuNPs. Each nanoparticle employed for the mass analysis was extracted randomly from collective TEM micrographs.

The mass distribution of the AuNPs and Fe_3_O_4_@Au NPs, which was crucial in order to construe the results related to signal amplification, was assessed by applying the software SPIP Mountains 8 to the TEM micrographs (Figure 2a,b). The nanoparticle of interest was considered and extracted from the collective TEM micrograph (I), the contour of the nanoparticles was detected and distinct by the background (II), then the particle surface (III) together with its 3D rendering (IV) were generated. The latter was employed to estimate the volume of the object. Once the volume of the nanoparticle had been estimated, its mass was obtained by multiplying by the density of the material. In Figure 2c the mass distribution of AuNPs (blue histogram) and of core-shell nanoparticles (red histogram) are compared. Each nanoparticle employed for the mass analysis was extracted randomly from collective TEM micrographs. The two mass distributions (Figure 2c) turned out to be unimodal distributions with the picks around the value 0.4 fg for Fe_3_O_4_@Au NPs (red histogram) and 0.63 fg for AuNPs (blue histogram). The standard deviations are respectively 0.2 fg and 0.09 fg. Thus, the masses of AuNPs and Fe_3_O_4_@Au NPs result to be of the same order of magnitude.

### 3.6. Experimental Setup

**Figure 3:**
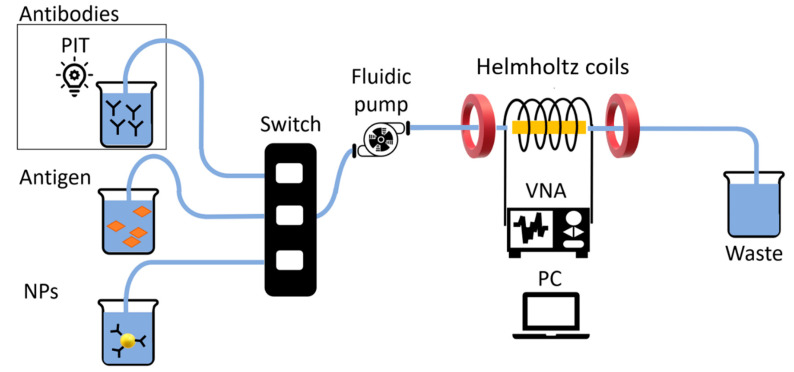
Schematic representation of the experimental setup. From left to right: the solution containing antibodies (activated via PIT), antigens and functionalized nanoparticles, fluidic channels and switch, fluidic (continuous) pump, Helmholtz coils, the ribbon enclosed by the cylindrical coil, the VNA connected to the PC and the flash out container (waste). A flow rate of 5 μL∙s^−1^ was used in order to ensure laminar flow over the magnetoelastic sensor. Resonance frequencies were continuously monitored and recorded by the analyzer (VNA) and computer system (PC). Finally, the waste analyte was collected in the flush out container for disposal.

The ME biosensor was mounted into a fluidic circuit by inserting it in a 3D printed acrylonitrile butadiene styrene (ABS) cell that was subsequently placed in a glass tube to be connected to a fluidic continuous pump (Figure 3). Two identical home-made Helmholtz coils were mounted at a distance equal to their radius and employed to produce a static and uniform magnetic field in the central region between them, where the glass tube, enclosing the ME sensor, was placed. A vector network analyzer (VNA) (E5071C ENA series, Keysight Technologies, California, USA) was connected to a home-made cylindrical coil wound around the glass tube containing the ME sensor. The cylindric single layer coil was made with 80 consecutive windings, using a copper wire (diameter of 0.1 mm), for a length of 8 mm and a diameter of 3.5 mm. The VNA operating using S-parameters, was employed to provide an AC field to excite the ribbon and monitor the reflected signal from the cylindric coil around the sensor. The reflection coefficient S_11_, i.e., the ratio between the amplitude of the reflected signal and the amplitude of the incident one, is commonly used to monitor the resonance frequency of a ME resonator [15]. In fact, S_11_ signal reaches its minimum at a frequency corresponding to the resonance frequency, $f_{0}$, of the sensor. 

The error of the experimental setup on a resonance frequency measure, extracted by fitting the signal obtained by the VNA, is of the order of 10^−2^ Hz, much smaller than the error related to stability fluctuation over time of $f_{0}$ (3 Hz as estimated in the next section).

### 3.7. Experimental Procedure

Before starting the sample injection, the fluidic circuits (flow rate of about 5 μL∙s^−1^) and the ME sensor were rinsed with MilliQ water. 

**Figure 4:**
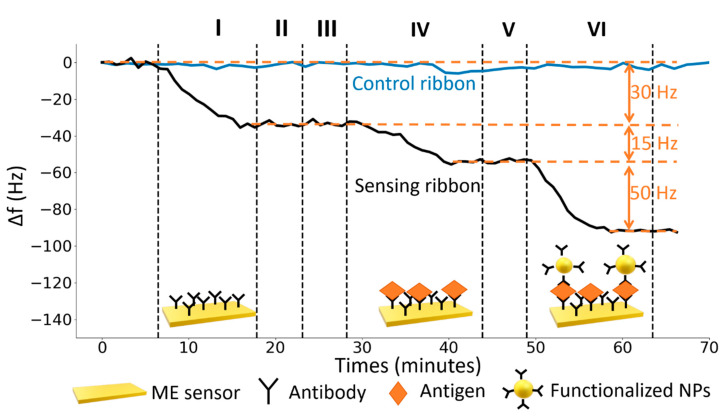
Typical dynamic response of the ME biosensor. On the *y* axis the shift of the resonance frequency $f_{0}$ due to the mass loading and on the *x* axis the time interval. The black line represents the response of the sensing ribbon in each of the following steps: (I) functionalization with a solution of 25 µg∙mL^−1^ of UV-activated antibodies, (II) rinse with MilliQ water, (III) flowing of bovine serum albumin solution (50 μg∙mL^−1^), (IV) flowing of target antigen solution (5 μg mL^−1^), (V) rinse with MilliQ water, (VI) amplification with core-shell magnetic NPs (Fe_3_O_4_@Au). The control ribbon, that was employed to estimate the noise level, is represented by a blue line.

A typical sensorgram reporting all the measurement steps is shown in Figure 4. In first step (I) a solution of 25 µg∙mL^−1^ of UV-activated antibodies (anti-human IgG produced in Goat) was conveyed to the cell for the surface functionalization (PIT). The decrease of the resonance frequency of the sensor makes evident that the functionalization took place correctly in just ten minutes. After the stabilization, the fluidic circuit was rinsed for five minutes with MilliQ water to remove the unbound Abs (II). Subsequently, a bovine serum albumin solution (50 μg∙mL^−1^) flowed into the fluidic circuit for five minutes to fill possible free space left by Abs on the gold surface (blocking, step III). In the step IV a solution of target antigen (Human IgG) flowed into the circuit for fifteen minutes. After the rinse (step V) a solution (1 mL) of functionalized core-shell nanoparticles was conveyed to the cell (step VI). As it can be noticed looking at Figure 4, the Fe_3_O_4_@Au NPs play an important role since the ME sensor response is eventually amplified by a factor slightly greater than three at this intermediate concentration. We highlight that all the steps were carried out until the equilibrium condition was reached thereby making more robust the whole approach. For each detection step the time to achieve a reliable stability was approximately five minutes, thus we carried out long term stability measurements (blue line in Figure 4), which we used to analyze the distribution of the means of the resonance frequency measured over intervals of five minutes. It turned up that the standard deviation (${\bar{\sigma }}_{f}$) of such a distribution was 1 Hz so that 3 Hz (3 SD) was used to determine the threshold to establish the occurrence of a signal (limit of detection).

## 4. Results

### 4.1. Comparison between AuNPs and Core-Shell NPs (Fe_3_O_4_@Au)

The idea of taking advantage of the magnetic interaction between magnetite nanoparticles and ME ribbons has already been employed in the past to detect bacteria [42]. In that case, Fe_3_O_4_ nanoparticles were modified by using chitosan, a linear polysaccharide, so that their surface was charged positively. In this way, in specific conditions, the nanoparticles bind to negatively charged bacteria as *E. coli* and therefore, thanks to magnetic attraction, they also bind to the surface of the ME sensor giving rise to a signal enhancement. This approach has several drawbacks. Firstly, the chitosan coating and the *E. coli* binding process are expensive and time consuming (several hours); and furthermore, the whole procedure must be carried out under controlled conditions, this preventing the application to complex matrices. Secondly, since the adhesion between bacteria and nanoparticles results from the electrostatic interaction between bacteria and the chitosan, it is expected that the specificity will be greatly compromised when other gram-negative bacteria are present in the sample. To circumvent such limitations, we functionalized the gold surface of Fe_3_O_4_@Au NPs with the antibodies targeting antigen, in this way achieving high specificity for the nanoparticle-antigen interaction. 

**Figure 5:**
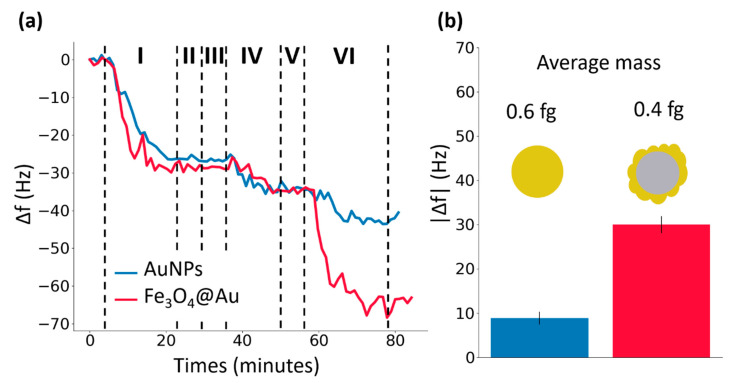
Comparison between the amplification effects due to AuNPs and core-shell magnetic NPs. (**a**) The blue and the red lines represent the response of the sensing ribbon to the following steps: (I) functionalization with 25 µg∙mL^−1^ of UV-activated antibodies (anti-human IgG) which causes a shift of the resonance frequency Δf ≃ 30 Hz; (II) rinse with MilliQ water; (III) flowing of bovine serum albumin solution (50 μg∙mL^−1^); (IV) exposure to the antigen solution (human IgG) which causes a shift Δf ≃ 10 Hz; (V) rinse with MilliQ water; (VI) amplification with AuNPs for the blue line and amplification with core-shell magnetic NPs for the red line. The former causes a shift Δf = 9 ± 1 Hz while the latter causes a shift Δf = 30 ± 2 Hz. (**b**) A direct comparison between the amplification of the response signal due to AuNPs (blue) and core-shell magnetic NPs (red).

Moreover, the magnetic core of the nanoparticles still played an important role since the (specific) nanoparticle-antigen bond is somehow catalyzed and enforced by the interaction between the magnetic dipole moment of the core-shell NPs and the strong local magnetic field. The occurrence of the latter process can be deduced by the results shown in Figure 5, in which the response signals obtained by exposing the sensor to 1 µg∙mL^−1^ of antigen solution and amplifying once with AuNPs (blue line) and once with core-shell NPs (red line) are reported. Even though their mass was smaller (Figure 2c), core-shell magnetic NPs were able to amplify the frequency shift by a larger amount (Figure 5) thereby demonstrating the higher sensitivity that can be achieved when the additional tool provided by magnetic moment is exploited.

### 4.2. Dose-Response Curve

**Figure 6:**
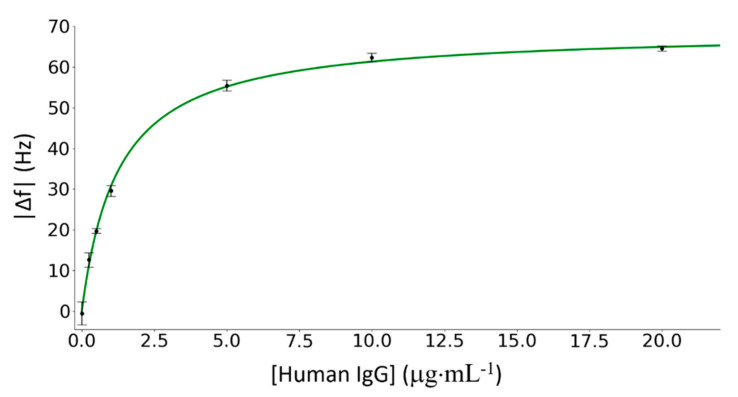
Dose–response curve, i.e., resonance frequency shifts Δf versus human IGg concentrations. Experimental data are fitted by Langmuir isotherm curve (Equation (5)). The range of tested concentrations varies from the zero concentration to 20 μg∙mL^−1^. Each concentration has been tested using different ribbons.

The dose-response curve is reported in Figure 6 together with the best fit of the experimental data provided by a Langmuir isotherm curve [43]:

(5) $$f\left(x\right)=a\frac{x}{x+C},$$

where *a* = 68.9 ± 0.5 Hz and *C* = 1.25 ± 0.04 μg mL^−1^ are the asymptotic value, and the concentration at which the frequency shift reaches the 50% of its maximal value, respectively. We carried out every experiment with a different ribbon obtaining coherent results. This is a strong confirm of the robustness of the experimental setup with respect to fluctuations related to differences in the fabrication process of ME sensors. The dose-response curve exhibits signal saturation at concentrations larger than 10 μg∙mL^−1^, thus showing that the ME immunosensor is able to provide a quantitative measurement over two decades. The error on each experimental point of the dose response curve was estimated by propagating the errors of the resonance frequency values in the equilibrium states before and after the amplification with core-shell NPs. The error of the resonance frequency of an equilibrium state was estimated as the standard deviation of the measured values in a time interval of five minutes. The limit of detection (LOD) was assessed inserting the error estimated in Section 3.6 ( $3\cdot {\bar{\sigma }}_{f}=\pm \;3\;\mathrm{Hz}$) in Equation (5) and turned up to be lower than 0.1 μg∙mL^−1^ (0.66 nM).

### 4.3. Specificity Test

**Figure 7:**
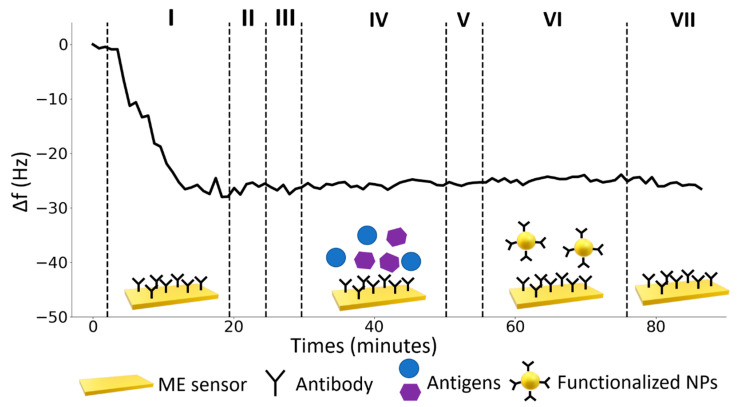
Sensor specificity. ME sensor responses to a mixed solution of rabbit IgG produced in sheep and mouse IgG produced in goat. The black line represents the response of the sensing ribbon in each of the following steps: (I) functionalization with a solution of 25 µg∙mL^−1^ of UV-activated antibodies, (II) rinse with MilliQ water, (III) flowing of bovine serum albumin solution (50 μg∙mL^−1^), (IV) flowing of Rabbit IgG produced in sheep and Mouse IgG produced in goat solution both at a concentration of 20 μg mL^−1^, (V) rinse with MilliQ water, (VI) amplification with core-shell NPs (Fe_3_O_4_@Au NPs), (VII) rinse with MilliQ water.

To ascertain the sensor specificity, the same experimental procedure was used to test the ME sensor with similar compounds. In the present case, we measured the response of the immunosensor to a mixture rabbit IgG produced in sheep and mouse IgG produced in goat at a concentration of 20 μg∙mL^−1^ each, whose sensorgram is shown in Figure 7. As it is clearly visible, only the shift resulting from the surface functionalization is visible, whereas no additional frequency shift is measured as a result of the presence of rabbit and mouse IgGs. This is true even when core-shell magnetic NPs are conveyed into the interaction cell (step VI). The high specificity of the immunosensor is largely a consequence of the excellent biorecognition properties of the antibodies.

## 5. Discussion

### 5.1. Estimation of the Magnetic Interaction among the ME Sensor and Core-Shell NPs

**Figure 8:**
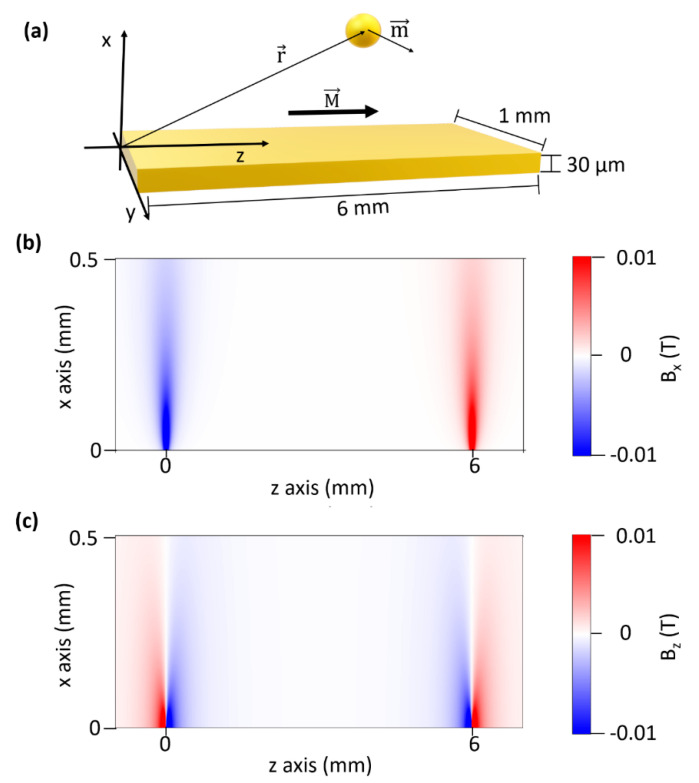
(**a**) Schematic representation of the interaction among the core-shell magnetic nanoparticle and the magnetoelastic ribbon. M is the magnetization of the ME ribbon, m is the magnetic moment of the core-shell nanoparticle and ***r*** indicates the position in the chosen reference system. (**b**,**c**).Intensity of the x and z components x of the magnetic induction field (*B_x_* and *B_x_*) in the symmetry plane x-z of the ribbon (y = 0.5 mm).

The magnetic force acting on each core-shell NP is

(6) $$F\left(r\right)=\nabla \left(m\cdot B_{tot}\right),$$

where m is the magnetic moment of a core-shell magnetic nanoparticle and $B_{tot}=B_{H}+B$ is the magnetic induction field generated by the Helmholtz coils ($B_{H}$) and the ME ribbon (***B***), respectively. The magnetic induction field produced by Helmholtz coils is directed along the *z* axis (Figure 8a) and can be considered uniform in the region around the ribbon. On the contrary, the magnetic induction field produced by the ME ribbon is not uniform and can be worked out by considering the ribbon as rectangularly shaped permanent magnet whose significant components can be written as [24,44]:

(7) $$B_{x}\left(x,y,z\right)=\frac{\mu_{0}M}{4\pi }\overset{2}{\underset{k=1}{\sum }}\overset{2}{\underset{m=1}{\sum }}{\left(-1\right)}^{k+m}\ln\left[\frac{\left(y-y_{1}\right)+{\left[{\left(x-x_{m}\right)}^{2}+{\left(y-y_{1}\right)}^{2}+{\left(z-z_{k}\right)}^{2}\right]}^{\frac{1}{2}}}{\left(y-y_{2}\right)+{\left[{\left(x-x_{m}\right)}^{2}+{\left(y-y_{2}\right)}^{2}+{\left(z-z_{k}\right)}^{2}\right]}^{\frac{1}{2}}}\right]$$

(8) $$B_{z}\left(x,y,z\right)=\frac{\mu_{0}M}{4\pi }\overset{2}{\underset{k=1}{\sum }}\overset{2}{\underset{n=1}{\sum }}\overset{2}{\underset{m=1}{\sum }}{\left(-1\right)}^{k+m+n}\tan^{-1}\left[\frac{\left(x-x_{n}\right)\left(y-y_{m}\right)}{\left(z-z_{k}\right){\left[{\left(x-x_{n}\right)}^{2}+{\left(y-y_{m}\right)}^{2}+{\left(z-z_{k}\right)}^{2}\right]}^{\frac{1}{2}}}\right],$$

where $\mu_{0}$ is the magnetic permeability of free space, $x_{1},\;x_{2}$, $y_{1},\;y_{2,\;}z_{1},\;z_{2}\;$ are the positions of the edges of the ribbon with respect to *x*, *y* and *z* axis (Figure 8a).

The magnetization M of the ribbon is oriented along the *z* axis and the value of $\mu_{0}M$ is approximately 0.2 T for Metglas [45]. The component *B_y_*, can be neglected since it is always much smaller than *B_x_* and *B_z_* while the latter are of the same order of magnitude and reach their maximum nearby the ribbons ends (Figure 8b,c). The dependence of *B_x_* and *B_z_* on x, y and z variables in Figure 8 suggests that the significant magnetic interaction is limited to a region with volume *S_xyz_* = 100 μm × 1 mm × 100 μm close to the ends of the ribbon. In this region $B_{H}$ is negligible with respect to *B_x_* and *B_z_* and does not contribute significantly to the nanoparticles’ magnetization. 

The magnetic moment, m, of a core-shell magnetic NP is the product of its magnetization, $M_{NP}$, and volume, $V_{m}=\frac{4}{3}\pi R_{m}^{3}$, where $R_{m}$ is the radius of its magnetic core, $m=M_{NP}\;V_{m}$. The volumetric magnetization is induced by the external magnetic induction field, $M_{NP}=\frac{∆\chi }{\mu_{0}}B$, where $∆\chi =\chi_{M_{NP}}-\chi_{water}\approx \chi_{M_{NP}}$ is the effective susceptibility of a magnetic nanoparticle with respect to the medium (water). Since the component of the magnetic induction field along the *y* axis is negligible, the magnetic moment of a core-shell NP lies on the x-z plane. The order of magnitude of the magnetic moment can be retrieved by the hysteresis cycles of Fe_3_O_4_ superparamagnetic nanoparticles provided by the seller and reported in Supplementary Materials Figure S1, also considering the effect of the gold shell that weakens magnetic properties [46]. The intensity of external magnetic induction field was high enough to induce significant magnetization, but only in the linear range of the magnetic response so the magnetic susceptibility could be considered constant in our case. 

Thus, the attractive magnetic force between the ribbon and a core-shell NP is:

(9) $$F_{x}\left(x,y,z\right)=-\frac{\partial \left(-m\cdot B\right)}{\partial x}=\frac{V_{m}∆\chi }{\mu_{0}}\left(2B_{x}\frac{\partial B_{x}}{\partial x}+2B_{z}\frac{\partial B_{z}}{\partial x}\;\right),$$

From the analysis of Equations (7) and (8), along the *x* axis, we have $B_{x}\approx B_{z}$, Figure 8b,c which entails $\frac{\partial B_{x}}{\partial x}\;\approx \;\frac{\partial B_{z}}{\partial x}\;\approx \;1\;T\cdot {\mathrm{mm}}^{-1}$; thus Equation (9) can be approximated as follows

(10) $$F_{x}\left(x,y,z\right)\approx 4\frac{V_{m}∆\chi }{\mu_{0}}\;B_{x}\left(x,y,z\right)\;\left[\frac{\partial B_{x}}{\partial x}\right]\;.$$

The force in Equation (10) bends the nanoparticles velocity field lines towards the ribbon thereby increasing the local density of the core-shell magnetic NPs. The order of magnitude of the bending can be estimated as the displacement induced by F_x_(x,y,z) acting on a nanoparticle in the region where the force is non-vanishing, i.e., the region S_xyz_ previously defined. The mean force ${\bar{F}}_{x}\;$ acting on core-shell magnetic NPs can be evaluated by averaging F_x_(x,y,z) in the region S_xyz_. According to Stokes’ law, for a spherical particle with radius r, the displacement caused by the mean force ${\bar{F}}_{x}$ is

(11) $$∆s_{m}\approx \mu \bar{F_{x}}t_{l},$$

where μ = (6πηr)^−1^ is the mobility, $t_{l}$ the time during which the interaction takes place. It should be noted that this approach is valid in the approximation that the motion is uniform along x, a condition well satisfied in our case since the limit velocity is reached within a very short time-interval (τ ≈ 10^−6^ s). The time $t_{d}$ in which the magnetic interaction takes place can be estimated as $t_{d}=d\cdot \;v_{flux}{}^{-1}\approx 1\;s$, where $v_{flux}\approx 100\;\mu m\cdot \;s^{-1}$ is the the longitudinal velocity of the liquid inside the channel and d≈100 μm the size of S_xyz_ along *z* axis. Eventually, Equation (11) provides Δs_m_ ≃30 μm that leads to an increase of the frequency collision between nanoparticles and the ribbon surface whereby more antigens (human IgGs) captured on the surface are ballasted by nanoparticles. 

The significance of such a bend arises from its comparison to the Brownian motion displacement [47]

(12) $$\Delta s_{B}=\sqrt{2Dt_{D}},$$

where *D* is the diffusion coefficient ($D=\mu k_{b}T\;\approx 10^{-11}{\;m}^{2}\cdot {\;s}^{-1}\;)$ and $t_{D}$ the diffusion time, which we can estimate by requiring $\Delta s_{B}\approx \Delta s_{m}$. Thus, from Equations (11) and (12), we obtain $t_{D}\approx 45$ s, a time much longer than the transit time of the core-shell NPs over the ME ribbon, which implies that the velocity field lines remain bent by magnetic force along the whole length of the ribbon. 

This view is confirmed by the analysis of the energy scales involved. The binding energy between the antibodies and the antigen is of the order of 1.6 10^−19^ J (1 eV), which is larger than the thermal energy at room temperature (k_B_T = 0.04 10^−19^ J = 0.025 eV). Interestingly, the potential well due to the magnetic induction field averaged over the region of interest S_xyz_, is of the order of k_B_T thus making consistent the description about the role played by the strong magnetic induction field at the edge of the ribbon in bending the velocity field lines and increasing the “effective” nanoparticle density, but without giving rise to any non-specific interaction with nanoparticles and the surface of the ribbon.

### 5.2. Future Research Direction and Applications

The ME-based biosensor presented here has proven to be a high-performing, rapid and reliable sensing technology that works effectively in water with a limit of detection (LOD) lower than 1 nM for an antigen as human IgG.

Compared with other sensing technologies, the ME-based sensor takes advantage of some features common to all magnetoelastic sensors. Indeed, they are simple in design and can be produced in small size using standard manufacturing procedures; they are very inexpensive so that the cost of manufacturing of these sensors is mainly the sensing element (i.e., antibody in this study), which is the same for all biological detection technologies. Therefore, these sensors can be used as disposable sensors. Furthermore, they are wireless, eliminating the need for direct physical contacts, thus favoring their use in real time applications such as detection in conductive liquids or in sealed and opaque containers, and biological experiments such as monitoring of blood flow chemistry. In addition to these features, the ME-based biosensor presented in this study, profits from the synergy with PIT allows a fast and efficient functionalization, contributing to the rapidity of the detection measure, which lasts approximately one hour. Moreover, PIT does not require complex chemical procedures, skilled personnel or laboratories, thus increasing the possibility of turning the biosensor into a portable device, suitable to perform real time and in situ detections. 

It should be mentioned that the possibility to functionalize surfaces in few minutes and not in laboratory conditions are key features that are not common to other experimental setups. Among all the possible functionalization strategies, self-assembled monolayers (SAMs) is currently one of the most widespread methods. Despite the advantages they offer in many applications, there are important drawbacks that should be considered to correctly evaluate their potential for on field applications. Firstly, despite SAMs on gold surfaces are often usually represented as compact monolayers, the realization of a well-assembled monolayer strongly relies on attention to details (i.e., the purity of the solutions used and the presence of even a low amount of contaminants [48]), that makes it not suitable for on field applications. On the other hand, an on field SAMs functionalization before detection would take several hours to be implemented. 

In this scenario, the advantages of combining PIT and the ME-based immunosensor are of great interest for environmental control and food safety applications, such as the detection of pesticides in water samples. Among the different pesticides, the glyphosate [(N-(phosphonomethyl)glycine)] is the most used herbicide worldwide. Due to its high solubility in water, there is a need of investigating its residual applications directly in fields for monitoring the contamination of aquatic environments [49]. Currently, there exist several high-sensitivity analytical techniques to detect pesticides, including glyphosate, mostly based on gas and liquid chromatography coupled with mass spectrometry [50]. However, their complexity prevents the opportunity of performing in situ and real-time analysis. Therefore, several types of biosensor-based technologies have emerged as promising tools for rapid on-site analysis of samples [51,52]. Among all types of biosensors, immunosensors techniques have already gained attention in the last decade, proving their effectiveness in this field. 

The stability in water of the ME biosensor, the rapidity of sample analysis and the possibility of turning the sensor into a portable device are essentials characteristics for future applications in detecting glyphosate. In addition, a sensitivity adequate to the legal limits of glyphosate concentrations is required. The European Union settled the maximum residue limit of glyphosate in drinking water to 0.1 μg∙L^−1^ (i.e., 0.5 nM), while in the United State of America the established limit is 700 μg∙L^−1^ (i.e., 4 μM) (Directive 2006/118/EC, Directive 2006/118/EC, USEPA. EPA 816-F-09-004). The ME immunosensor shows a LOD of 0.66 nM for the tested antigen, which is well below the maximum concentration allowed in the United State of America and of the same order of magnitude of the European one, thereby suggesting future applications to the detection of pesticides in water.

## 6. Conclusions

In this paper a high-performance magnetoelastic (ME) biosensor for wireless recognition of antigens in liquid is presented. The surface of the biosensor is functionalized with antibodies by using a very effective immobilization technique (photochemical immobilization technique, PIT). 

The performances of the device were tested with human IgG and different ribbons were used for each measurement. An innovative signal amplification method has also been introduced which exploits core-shell magnetic nanoparticles (Fe_3_O_4_@Au) so to exploit both the magnetic effect due to the core and the gold nanoflowers on the surface, the latter being effective in tethering antibodies by PIT. The results obtained with magnetic nanoparticles have been compared with those obtained with gold nanoparticles, showing that the magnetic character of the former plays a crucial role for improving the performance. 

PIT was used here for the first time to functionalize a ME biosensor, allowing us to carry out the whole measurement in about 1 h. The limit of detection (LOD) lower than 1 nM paves the way to its applications to environmental and food safety for on field measurements.

## Supplementary Materials

The following are available online at https://www.mdpi.com/2079-4991/10/8/1526/s1, Figure S1: Room-temperature M-H curve of the magnetite samples [supplied by the nanoparticles manufacturer (Ocean Nano Tech, LLC)] measured by cycling the external magnetic field between −14,000 Oe and 14,000 Oe. This magnetization curve shows a very small hysteresis behavior for the samples and exhibits small values of coercive field and remnant magnetization. This indicates that the nanoparticles can safely be considered as superparamagnetic.
