# Supplementary material for: Core-Shell Magnetic Nanoparticles for Highly Sensitive Magnetoelastic Immunosensor
Source: Nanomaterials (Basel). 2020 Aug 4;10(8):1526. doi: 10.3390/nano10081526 (PMC7466411; doi:10.3390/nano10081526)
Supplement: Supplementary file 1 [file nanomaterials-10-01526-s001.pdf]

nanomaterials

MDPI

*SUPPLEMENTARY MATERIALS*

# Core-shell Magnetic Nanoparticles for highly sensitive Magnetoelastic Immunosensor

**Raffaele Campanile [1,2], Emanuela Scardapane [1,2], Antonio Forente [1], Carmine Granata [3], Roberto Germano [2], Rocco Di Girolamo [4], Antonio Minopoli [1], Raffaele Velotta [1,3], Bartolomeo Della Ventura [1,3], Vincenzo Iannotti [1,5,*]**

[1] Department of Physics "E. Pancini", University of Naples Federico II, Via Cintia 26, I-80126 Napoli, Italy; raffaele.campanile@unina.it (R.C); emanuela.scardapane@unina.it (E.S); a.forente@studenti.unina.it (A.F); antonio.minopoli2@unina.it (A.M); rvelotta@unina.it (R.V); Bartolomeo.dellaventura@unina.it (B.D.V).

[2] PROMETE Srl, CNR Spin off, Piazzale Tecchio, 45 80125 Napoli (NA), Italy germano@promete.it (R.G.).

[3] Institute of Applied Sciences and Intelligent Systems of the National Research Council (CNR-ISASI), Via Campi Flegrei 34, I-80078 Pozzuoli (NA), Italy; c.granata@isasi.cnr.it (C.G.).

[4] Department of Chemistry, University of Naples "Federico II", Via Cintia 26, I-80126 Napoli, Italy; rocco.digirolamo@unina.it (R.D.G).

[5] Institute for Superconducting, Oxides and other Innovative Materials and Devices of the National Research Council (CNR-SPIN), Piazzale V. Tecchio 80, I-80125 Napoli, Italy

* Correspondence: viannotti@unina.it ; Tel.: 081 7682419 (V.I.)

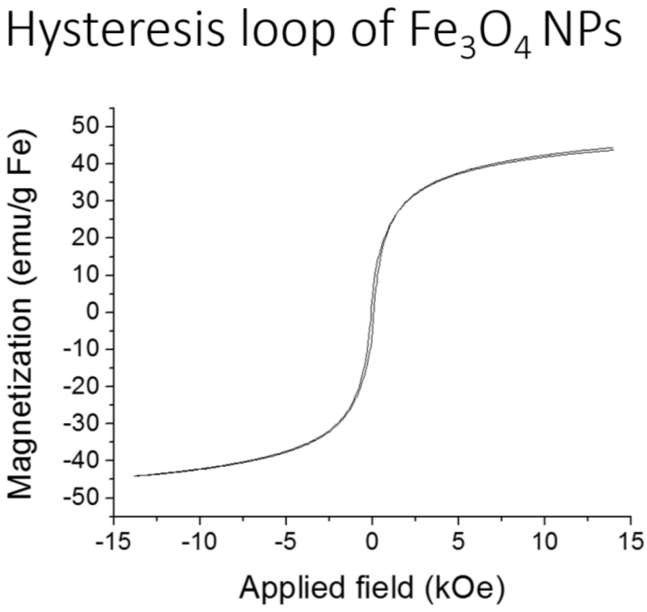


Figure S1. Room-temperature M-H curve of the magnetite samples [supplied by the nanoparticles manufacturer (Ocean Nano Tech, LLC)] measured by cycling the external magnetic field between −14000 Oe and 14000 Oe. This magnetization curve shows a very small hysteresis behavior for the samples and exhibits small values of coercive field and remnant magnetization. This indicates that the nanoparticles can safely be considered as superparamagnetic.
